# A Quantitative Structure-Property Relationship (QSPR) Study of Aliphatic Alcohols by the Method of Dividing the Molecular Structure into Substructure

**DOI:** 10.3390/ijms12042448

**Published:** 2011-04-07

**Authors:** Fengping Liu, Chenzhong Cao, Bin Cheng

**Affiliations:** 1 School of Chemistry and Chemical Engineering, Hunan University of Science and Technology, Xiangtan, Hunan 411201, China; E-Mails: lfp6907@163.com (F.L.); chengbin_1001@163.com (B.C.); 2 Key Laboratory of Theoretical Chemistry and Molecular Simulation of Ministry of Education, Hunan University of Science and Technology, Xiangtan, Hunan 411201, China

**Keywords:** quantitative structure property relationship, aliphatic alcohols, boiling points, *n*-octanol-water partition coefficient, water solubility, retention indices

## Abstract

A quantitative structure–property relationship (QSPR) analysis of aliphatic alcohols is presented. Four physicochemical properties were studied: boiling point (BP), *n*-octanol–water partition coefficient (lg *P*_OW_), water solubility (lg *W*) and the chromatographic retention indices (RI) on different polar stationary phases. In order to investigate the quantitative structure–property relationship of aliphatic alcohols, the molecular structure ROH is divided into two parts, R and OH to generate structural parameter. It was proposed that the property is affected by three main factors for aliphatic alcohols, alkyl group R, substituted group OH, and interaction between R and OH. On the basis of the polarizability effect index (PEI), previously developed by Cao, the novel molecular polarizability effect index (MPEI) combined with odd-even index (OEI), the sum eigenvalues of bond-connecting matrix (SX_1CH_) previously developed in our team, were used to predict the property of aliphatic alcohols. The sets of molecular descriptors were derived directly from the structure of the compounds based on graph theory. QSPR models were generated using only calculated descriptors and multiple linear regression techniques. These QSPR models showed high values of multiple correlation coefficient (*R >* 0.99) and Fisher-ratio statistics. The leave-one-out cross-validation demonstrated the final models to be statistically significant and reliable.

## 1. Introduction

Quantitative structure–property relationships (QSPR) remain the focus of many studies aimed at the modeling and prediction of physicochemical and biological properties of molecules. A powerful tool to help in this task is chemometrics, which uses statistical and mathematical methods to extract maximum information from a data set.

QSPR uses chemometric methods to describe how a given physicochemical property varies as a function of molecular descriptors describing the chemical structure of the molecule. Thus, it is possible to replace costly biological tests or experiments of a given physicochemical property (especially when involving hazardous and toxically risky materials or unstable compounds) with calculated descriptors, which can in turn be used to predict the responses of interest for new compounds. Chemometrics has provided new insight into the philosophy and theory behind QSPR modeling [1,2]. It has been used to estimate properties such as density, boiling point, solubility, *n*-octanol–water partition coefficient, Henry’s law constant and vapor pressure of chemicals. QSPR has received significant contributions from various research schools [3–8]. Various quantitative structure–property relationship (QSPR) models have been proposed for estimating the properties of a series of aliphatic alcohols [9–12].

The basic strategy of QSPR is to find an optimum quantitative relationship, which can be used for the prediction of the properties of compounds, including those unmeasured. It is obvious that the performance of QSPR model mostly depends on the parameters used to describe the molecular structure. Many efforts have been made to develop alternative molecular descriptors which can be derived using only the information encoded in the chemical structure. Much attention has been concentrated on “topological indices” derived from the connectivity and composition of a molecule which have made significant contributions in QSPR studies. Topological index has advantages of simplicity and quick speed of computation and so attracts the attention of scientists. Topological descriptors can explain most of the property modeled, as shown by some researchers [13].

In order to investigate the quantitative structure–property relationship of aliphatic alcohols, the molecular structure ROH is divided into two parts, R and OH to generate structural parameter. We proposed that the property is affected by three main factors for aliphatic alcohols, alkyl group R, substituted group OH, and interaction between R and OH. Due to the simplicity and efficiency of graph-theoretical approaches, our group recently introduced a set of novel topological indices to establish the quantitative relationships between the physicochemical properties and molecular structure for organic compounds [14–17]. On the basis of the polarizability effect index (PEI) previously developed by Cao, the novel molecular polarizability effect index (MPEI) combined with odd-even index (OEI), the sum eigenvalues of bond-connecting matrix (SX_1CH_) previously developed in our team, were used to predict the property of aliphatic alcohols.

The main goal of the present study was to obtain QSPR models of the boiling point (BP), *n*-octanol–water partition coefficient (lg *P*_OW_), water solubility (lg *W*) and the chromatographic retention indices (RI) for aliphatic alcohols using only calculated descriptors. At first, the generated numerical descriptors that encode structural information for the compounds in the data set were calculated. Then, multiple linear regression statistical analysis was used to build the QSPR models. In these models, no physical property parameter was used so that prediction could be carried out directly from molecular structure.

## 2. Methodology

The QSPR study of these aliphatic alcohols was performed in four fundamental stages: (1) Selection of data set; (2) generation of molecular descriptors; (3) multiple linear regression statistical analysis; and (4) model validation techniques. The descriptive power of the model was characterized by use of the statistical data multiple correlation coefficient (*R*), Fisher ratio (*F*), and standard derivation (*s*). Model applicability was further examined by plotting predicted data against experimental data for all the compounds.

All calculations were run on a Pentium IV personal computer with XP as operating system. Computation of the descriptors was performed using Matlab 6.5 programs. The Origin program packages were employed for regression analysis [18].

### 2.1. Data Set

Alcohols are toxic materials and thus represent dangerous environmental pollutants especially in the case when a mishap happens and accidentally large quantities of alcohols pollute the environment. Alcohols are also technologically important materials and are used in the manufacture of a large number of products. In this work, 58 aliphatic alcohols were studied. The corresponding experimental data (boiling points at 1 atm) were obtained from the literature [19]. The water solubility (lg *W*) and *n*-octanol/water partition coefficients (lg *P*_OW_) of the alcohols were taken from the literature [20]. The data sets of the Kovats retention indices were taken from the literature [21]. Kovats retention indices of the molecules were obtained on six different stationary phases of low to medium polarity (SE-30, OV-3, OV-7, OV-11, OV-17 and OV-25). All of these data are in agreement with a standard source.

### 2.2. Definition of the Topological Indices

Descriptors encoding significant structural information are used to present the physicochemical characteristics of compounds to build the relationship between structure and property in this study. According to the basic factors that influence the property of the aliphatic alcohols, such molecular descriptors: the molecular polarizability effect index (MPEI) connecting to the polarizability of the molecule and the intramolecular action of the solute, the odd–even index (OEI) which reflects the size of the molecule and the connection of each atom, the sum eigenvalues of every H–C bond adjacency matrix(SX_1CH_) connecting to the property of the chemical bond, have been generated to build the QSPR model. The index OEI and SX_1CH_ reflect the property of apolar R group and represent the R contribution to the physicochemical properties to be predicted. The MPEI index reflects the property of polar OH group and represents the OH contribution, and R/OH interaction contribution. A complete list of the compounds names and the calculated values of the molecular descriptors appearing in the QSPR models are summarized in Tables 3, 5 and 6.

#### 2.2.1. The Odd–Even Index OEI

**Figure 1.**
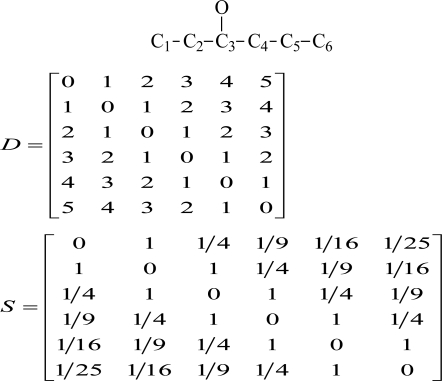
The hydrogen-suppressed molecular graph of 3-hexanol.

Odd–even index has been defined for the alkane molecule in our previous paper [14], which reflects the size of the molecule and the connection of each atom. The index is restated briefly as follows:

(1) $$\text{OEI }=\;\sum_{i=1}^{N}\sum_{j\neq 1}^{N}\left[{\left(-1\right)}^{D_{ij}-1}\;S\right]$$

where *N* is the number of vertices in molecular graph and *S* is the derivative matrix from distance matrix *D*. The elements of *S* are the squares of the reciprocal distances (*D_ij_*)^−2^, *i.e.*, $S\;=\;[1/D_{ij}^{2}]$ (when *i* = *j*, let $1/D_{ij}^{2}\;=\;0$). Taking 3-hexanol as an example to illustrate the calculation of OEI: First, we convert the structure of the molecule into that of the corresponding hexane. Figure 1 shows the hydrogen-suppressed molecular graph of 3-hexanol, where the numbers are the random numberings of each vertex. Then, we use matrices *D* to represent *D_ij_* of the molecule.

According to Equation (1), OEI is computed as follows:

$$\text{OEI }=\text{ 1}\times 10\;+\;\left[-\frac{1}{4}\right]\times 8+\left[\frac{1}{9}\right]\times 6+\left[-\frac{1}{16}\right]\times 4+\left[\frac{1}{25}\right]\times 2\;=\;8.4967$$

#### 2.2.2. The Molecular Polarizability Effect Index MPEI

In the preceding paper [16], the polarizability effect index (PEI) for alkyl groups of alkane molecules has been developed and calculated. It quantitatively indicates the relative proportion polarizability effect of the alkyl groups. The PEI values of some normal alkyls and the increments ΔPEI are listed in Table 1. As with aliphatic alcohols, the contribution of the property arising from relative proportion polarizability effect of alkyl groups is expressed as:

(2) PEI = ∑[∑(ΔPEI)]

where ΔPEI is the polarizability effect index increment of *i*th essential unit and can be directly taken from Table 1.

For the aliphatic alcohol molecules, the substituent *R* contains other atoms besides carbon and hydrogen, *α_i_* is no longer a constant and Equation (2) will not work well. It needs to be modified. Here, we use Equation (3) to evaluate the stabilizing energy caused by the polarizability effect for a substituent *R*:

(3) $$E\;(R)\;=\;K_{m}\sum \alpha_{i}\;\left[\Delta \text{PEI(}R_{i})\right]$$

where *K_m_* = −*q*^2^/(2Dl^4^), *α_i_* is the polarizability (unit 10^−24^ cm^3^) of the *i*th atom in substituent R. Some atomi *α_i_* values are listed in Table 2. Because *K_m_* is a constant, this work only calculates the term Σ*α_i_* (ΔPEI) of Equation (3). Take the sum of Σ*α_i_* (ΔPEI) for all groups in a molecule as the molecular polarizability effect index (MPEI) and MPEI is expressed as [16]:

(4) $$\text{MPEI }=\;\sum [\sum \alpha_{i}\;(\Delta \text{PEI)}]$$

The molecule of 2-methyl-1-propanol is taken as an example to illustrate the calculation of the molecular polarizability effect index.

**Figure 2.**
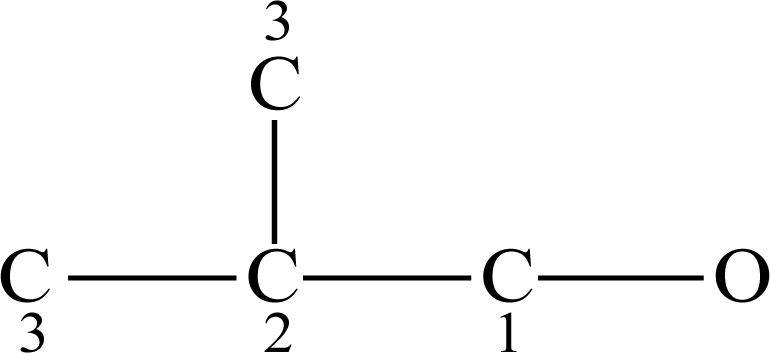
The hydrogen-suppressed molecular graph of 2-methyl-1-propanol.

Figure 2 shows its hydrogen-suppressed molecular graph, where the numbers are the numberings of each carbon atom according to its distance to the hydroxide radical. Take the carbon atom connecting the hydroxide radical as the beginning to calculate the MPEI index as below:

$$\begin{array}{lll} \text{MPEI} & = & 1\text{.76 }+\;\text{(0}\text{.802 }+\text{ 0}\text{.6668}\times 2\;+\text{ 1}\text{.76)}\times 0\text{.140526 }+\text{ (0}\text{.6668}\times 2\;+\text{ 1}\text{.76}\times 2)\times 0\text{.048132}\\ & & +\text{ 0}\text{.6668}\times 6\times 0\text{.023503 }=\text{ 2}\text{.6351} \end{array}$$

#### 2.2.3. Eigenvalues of Bond-Connecting Matrix (SX_1CH_)

**Figure 3.**
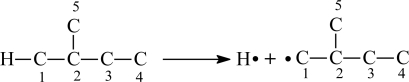
The breaking of the H–C bond of 2-methylbutane molecule.

Recently, we introduced the X_1CH_ index to evaluate bond dissociation energy for the alkane molecule [15]. Here, we also convert the structure of the aliphatic alcohol molecule to that of the corresponding alkane. Now, we consider the molecule of 2-methyl-1-propanol, the corresponding alkane is 2-methylbutane. If H atom connects with the *i*th carbon atom (C*_i_*), when the H*–*C*_i_* bond is broken, two radicals H and R*_i_* will be formed (Figure 3).

According to the calculation method of PEI of alkyl in paper [16] and values in Table 1, we can calculate the PEI for two radicals above as follows:

H: PEI_H_ = 0
R_1_: PEI_1_ = 1.2122+ 0.0481= 1.2603

Then, PEI_H_ and PEI_1_ were used as the main diagonal elements to build the bonding adjacency matrix B_CH_ of H*–*C_1_ bond:

$${\text{B}}_{\text{CH}}\;=\;\left[\begin{array}{cc} {\text{PEI}}_{\text{H}} & 1\\ 1 & {\text{PEI}}_{\text{l}} \end{array}\right]\;=\;\left[\begin{array}{cc} 0 & 1\\ 1 & 1.2603 \end{array}\right]$$

The off-diagonal element “1” in matrix means that H atom and C_1_ are connected with each other, *i.e.*, they are adjacent. Solving matrix B_CH_ by computer, we got two eigenvalues X_1CH_ = −0.5518 and X_2CH_ = 1.8121 (let X_1CH_ < X_2CH_). The eigenvalues of every H–C bond adjacency matrix in a molecule are also calculated with the same method. Finally, taking sum of X_1CH_ of all B_CH_ respectively, we got parameters SX_1CH_, in other words, let SX_1CH_ = ∑X_1CH_. For 2-methylbutane, there are:

$${\text{SX}}_{1\text{CH}}\;=\;\sum {\text{X}}_{1\text{CH}}\;=\;6\times \text{(}-0\text{.5518) }+\;\text{(}-0\text{.5061) }+\text{ 2}\times (-0\text{.5255)}\;+\text{ 3}\times \text{(}-0\text{.5576) }=\;-6\text{.5407}$$

## 3. Results and Discussion

Multiple linear regression analysis using the novel MPEI, OEI and SX_1CH_ indices is performed for the development of the final QSPR model.

### 3.1. Quantitative Structure-Retention Relationship (QSRR) Model for Alcohols on Stationary Phases of Different Polarity

After calculation of the descriptors (Table 3) of alcohols molecule, multiple linear regression analysis using the novel MPEI, OEI, SX_1CH_ indices is performed for the development of the final QSRR model for each stationary phase separately. Specifications of the best models found for describing the RI values of alcohols on the six stationary phases are given in Table 4. It can be seen that the equations represent excellent QSRR models judging from high *R* and low *s* values. Also, the *F* values show a high degree of statistical credibility and are indicative of an excellent fit of the models to the experimental RI values.

In order to validate the models obtained, the leave-one-out test was performed. The results for the models are shown in Table 4. As shown, in all cases, cross-validated correlation coefficient is only slightly less than the corresponding value of the full model.

### 3.2. Quantitative Structure-Property Relationship (QSPR) Model for BP of the Alcohols

Boiling point is important for the characterization and identification of a compound. It also provides an indication of the volatility of a compound. It is intuitively evident that boiling point is critically influenced by two characteristics of a molecule: first the molecular weight and, second, the intermolecular attractive forces between molecules. Multiple linear regression analysis using the novel MPEI, OEI indices is performed for the development of the final two-parameter QSPR model in the form of Equation (5). Of the two parameters in the model, the OEI index addresses the first, and the MPEI addresses the second.

(5) BP = 187.7855 + 12.8416OEI − 53.8368MPEI

*R* = 0.9928; *s* = 4.3; *F* = 1885.3; *n* = 58; *R_cv_* = 0.9918; *s_cv_* = 4.5.

**Figure 4.**
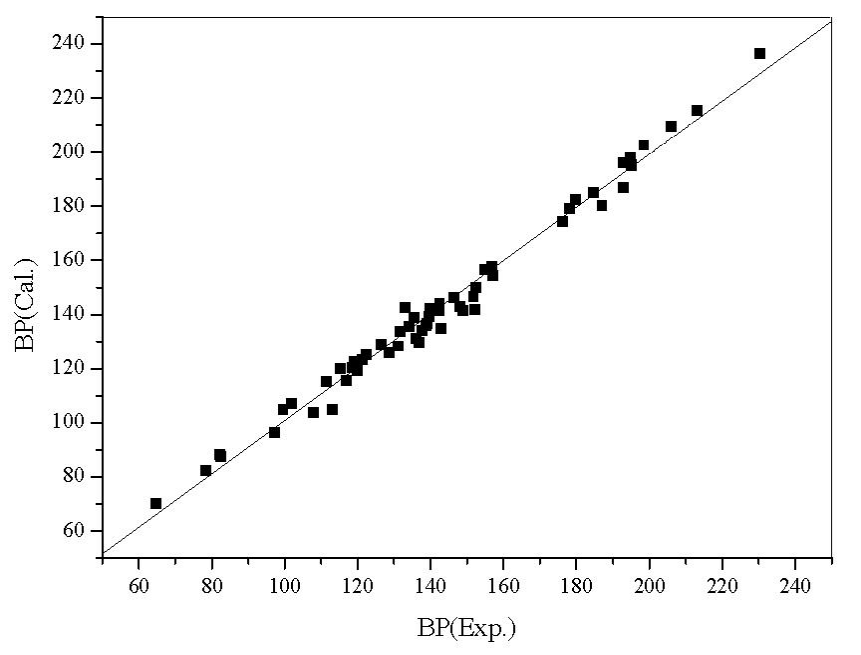
The plot of the calculated *vs.* the experimental BP for 58 aliphatic alcohols.

The two parameter QSPR equation reflects quantitatively the well known fact that the boiling point of a compound depends on the mass of its molecules and their tendency to stick together. The calculated BP is shown in Table 5 and plotted against the experimental values in Figure 4.

### 3.3. Quantitative Structure-Property Relationship (QSPR) Models for Water Solubility (lg *W*), *n*-Octanol/Water Partition Coefficients (lg *P*_OW_) of the Alcohols

Physicochemical properties of micropollutants, such as water solubility (lg *W*) and *n*-octanol/water partition coefficient (lg *P*_OW_), play a major role in determining the distribution and fate of organic contaminants in the global environments and have been used for assessing environmental partition and transport of organic substances. The compounds used in this study contain 58 alcohols. With the aid of a computer program, the best model is obtained as follows:

$$\text{lg }W\;=\;-0\text{.5370 }-\text{ 1.2930MPEI }-\text{ 0}{\text{.5950SX}}_{1\text{CH}}$$

*R* = 0.9942; *s* = 0.19; *F* = 2176.9; *R_cv_* = 0.9932; *s_cv_* = 0.20; *n* = 58.

$$\text{lg }P_{\text{OW}}\;=\;-\text{ 1.0271 }-\text{ 0.7113MPEI }-\text{ 0}{\text{.5531SX}}_{1\text{CH}}$$

*R* = 0.9959; *s* = 0.15; *F* = 3306.4; *R_cv_* = 0.9954; *s_cv_* = 0.15; *n* = 58.

Two models are validated to be statistically significant by the leave-one-out cross-validation. The calculated and experimental lg *W* and lg *P*_OW_ of alcohols along with topological descriptors are listed in Table 6.

**Figure 5.**
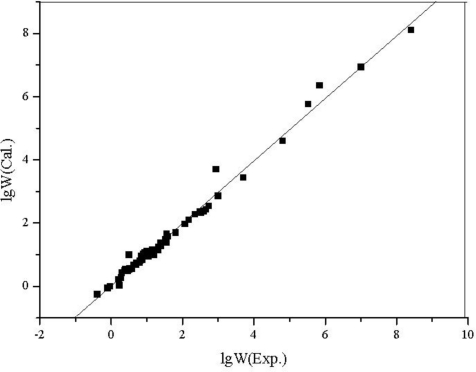
The plot of the calculated *vs.* the experimental lg *W* for 58 aliphatic alcohols.

**Figure 6.**
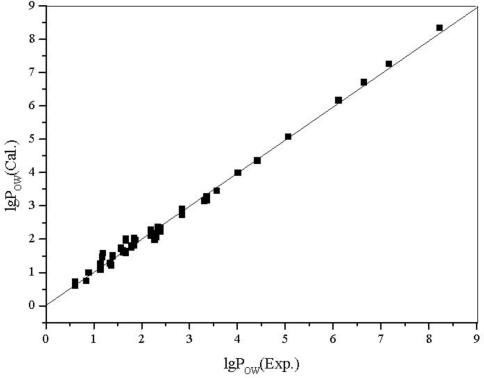
The plot of the calculated *vs.* the experimental lg *P*_OW_ for 58 aliphatic alcohols.

The plot of calculated values *versus* observed values of lg *W* and lg *P*_OW_ is shown in Figure 5 and Figure 6, respectively.

In the three models, the proposed index OEI and SX_1CH_ were generated on the basis of the aliphatic part of the molecule and represent the R contribution to the physicochemical properties to be predicted. The MPEI index was introduced not only taking into account the presence of OH group, but also the polar OH contribution and apolar R group/polar OH interaction contribution to the predicted physicochemical properties. The property of alcohols is influenced by the intermolecular forces and MPEI index connecting to the polarizability of the molecule and the intramolecular action of the solute. So, in the three different models, MPEI index is significant.

Most of QSPR research only investigates one or a few properties of correlation with some parameters or descriptors. In this paper, we have obtained good correlations between OEI, MPEI, SX_ICH_ and the many properties of alcohols.

From the results above, all of the correlation coefficients (*R*) are greater than 0.99, every regression equation has high *F* and low *s*; from the figures, the calculated values are very close to the experimental ones, there is no large deviation in all estimated values, and the statistical validity of the models are verified by the leave-one-out cross validation technique.

It appears that models based on these properties are simpler, but it is important to remember that the experimental data of these properties are not always available. Furthermore, their predicted data could be subject to high variability due to the selected QSPR calculation method.

## 4. Conclusion

In this study, the novel topological indices: MPEI, OEI and SX_1CH_ based on graph theory by dividing the molecular structure into substructure, were used to correlate with boiling point (BP), octanol–water partition coefficient (lg *P*_OW_), water solubility (lg *W*) and the chromatographic retention indices (RI) on different polar stationary phases. Descriptors appeared in these models coding the chemical structure effectively and simply, providing enough information related to the molecular structure and molecular properties. The proposed models have good stability, robustness and the predicted values from MLR method are close to the experimental values, which demonstrates the ability of these descriptors to give prediction. The leave-one-out cross-validation technique used in the study ensures the models performed as stably and reliably as possible. The correlation equations and descriptors are expected to be used for the prediction of physicochemical properties for diverse aliphatic alcohols in cases where the physicochemical indices are not readily available. This paper opens a new insight and may lead to the exploration of a novel way for QSPR study of alcohols.

## Figures and Tables

**Table 1.** ΔPEI values of the *i*th essential unit in alkyl substituent.

***N****_i_*	**ΔPEI**	***N****_i_*	**ΔPEI**	***N****_i_*	**ΔPEI**	***N****_i_*	**ΔPEI**
1	1.00000	6	0.009052	11	0.002375	16	0.001073
2	0.140526	7	0.006388	12	0.001972	17	0.000945
3	0.048132	8	0.004748	13	0.001628	18	0.000838
4	0.023503	9	0.003666	14	0.001421	19	0.000749
5	0.013800	10	0.002196	15	0.001229	20	0.000673

**Table 2.** *α_i_* values of some atoms [22].

**Atom**	**H**	**C**	**S**	**O**	**F**	**Cl**	**Br**	**I**
*α_i_*	0.6668	1.76	2.90	0.802	0.557	2.18	3.05	5.34

**Table 3.** Retention indices on different polar stationary phases of saturated alcohols and the topological descriptors values used in the QSRR models.

		**Retention Indices**						**Descriptors**		

**No.**	**Alcohol**	**SE-30**	**OV-3**	**OV-7**	**OV-11**	**OV-17**	**OV-25**	**OEI**	**MPEI**	**SX_1CH_**
1	1-butanol	650	672	702	725	748	792	5.2222	2.5887	−6.5340
2	1-hexanol	856	881	907	935	959	1003	8.4967	2.6446	−8.5424
3	1-heptanol	960	985	1010	1038	1062	1104	10.1183	2.6611	−9.5424
4	2-butanol	586	607	633	656	675	711	5.2222	2.7854	−6.5407
5	2-pentanol	689	711	735	756	777	811	6.8194	2.8386	−7.5453
6	3-pentanol	689	708	733	756	777	808	6.8194	2.8850	−7.5440
7	3-hexanol	785	807	830	853	878	904	8.4967	2.9383	−8.5434
8	3-heptanol	886	909	929	955	975	1008	10.1183	2.9715	−9.5414
9	4-heptanol	880	904	924	946	968	999	10.1183	2.9916	−9.5392
10	2-methyl-2-butanol	628	652	674	692	709	738	6.4444	3.0353	−7.5706
11	2-methyl-2-hexanol	822	848	862	884	904	930	9.6739	3.1217	−9.5480
12	2-methyl-2-heptanol	920	944	961	982	1001	1026	11.2400	3.1444	−10.5425
13	2-methyl-3-hexanol	858	876	897	920	939	969	9.6739	3.0379	−9.5407
14	3-methyl-1-butanol	725	747	771	798	817	855	6.4444	2.6420	−7.5453
15	4-methyl-1-pentanol	827	849	876	902	923	960	7.9167	2.6551	−8.5469
16	2-ethyl-1-hexanol	1019	1046	1067	1092	1116	1156	11.5178	2.7975	−10.5296
17	3-ethyl-3-pentanol	853	876	898	920	939	974	9.9583	3.2345	−9.5358
18	2,2-dimethyl-3-pentanol	814	834	855	874	890	919	8.5139	3.0843	−9.5556
19	2,2-dimethyl-3-hexanol	906	926	944	962	977	1004	10.3511	3.1375	−10.5326
20	1-propanol	544	574					3.5000	2.5354	−5.5244
21	1-pentanol	751	777		806	856	900	6.8194	2.6219	−7.5404
22	2-pexanol	787	811	835		878	914	8.4967	2.8718	−8.5469
23	2-methyl-1-propanol	612	641	654		680	740	4.5000	2.6351	−6.5407
24	2-methyl-2-pentanol	726	748	767		801	827	7.9167	3.0886	−8.5515
25	2-ethyl-1-butanol	834	857		907	928		8.2639	2.7417	−8.5400

**Table 4.** Statistical results of MLR models for RI based on six stationary phases with topological indices.

**Stationary Phase**	**Regression Equation**	**Statistics**					
		***R***	***s***	***F***	***R****_cv_*	***s****_cv_*	***n***
SE-30	RI = 714.1971 – 53.1823SX_1CH_	0.9963	11.2	942.1	0.9943	12.8	25
	–231.145MPEI + 34.62949OEI						
OV-3	RI =756.8884 – 52.1502 SX_1CH_	0.9963	11.2	936.4	0.9942	12.8	25
	–236.867MPEI + 35.3456OEI						
OV-7	RI = 798.1506 – 47.8311SX_1CH_	0.9953	12.3	638.7	0.9922	14.3	22
	–238.579MPEI + 37.97237OEI						
OV-11	RI = 858.8273 – 43.7851SX_1CH_	0.9938	13.8	453.1	0.9891	16.4	21
	–249.092MPEI + 41.39177OEI						
OV-17	RI = 941.0954 – 35.5304SX_1CH_	0.9940	13.6	547.6	0.9899	16.1	24
	–263.948MPEI + 47.63748OEI						
OV-25	RI = 1053.736 – 37.8516 SX_1CH_	0.9922	15.6	402.5	0.9871	18.3	23
	–292.817MPEI + 45.8317OEI						

**Table 5.** Experimental and calculated boiling points (BP) of 58 saturated alcohols and the topological descriptors values used in the QSPR model.

**No.**	**Alcohol**	**OEI**	**MPEI**	**BP (Exp.)**	**BP (Cal.)**	**ΔBP**
1	methanol	0.0000	2.1859	64.7	70.1	−5.4
2	ethanol	2.0000	2.4358	78.3	82.3	−4.0
3	1-propanol	3.5000	2.5354	97.2	96.2	1.0
4	1-butanol	5.2222	2.5887	117.0	115.5	1.5
5	1-pentanol	6.8194	2.6219	137.8	134.2	3.6
6	1-hexanol	8.4967	2.6446	157.0	154.5	2.5
7	1-heptanol	10.1183	2.6611	176.3	174.5	1.8
8	1-octanol	11.7808	2.6736	195.2	195.1	0.1
9	1-nonanol	13.4120	2.6835	213.1	215.5	−2.4
10	1-decanol	15.0680	2.6914	230.2	236.4	−6.2
11	2-propanol	3.5000	2.6857	82.3	88.1	−5.8
12	2-butanol	5.2222	2.7854	99.6	104.9	−5.3
13	2-pentanol	6.8194	2.8386	119.0	122.5	−3.5
14	2-hexanol	8.4967	2.8718	139.9	142.3	−2.4
15	2-octanol	11.7808	2.9110	179.8	182.4	−2.6
16	2-nonanol	13.4120	2.9235	198.5	202.6	−4.1
17	3-pentanol	6.8194	2.8850	115.3	120.0	−4.7
18	3-hexanol	8.4967	2.9383	135.4	138.7	−3.3
19	3-heptanol	10.1183	2.9715	156.8	157.7	−0.9
20	4-heptanol	10.1183	2.9916	155.0	156.7	−1.7
21	3-nonanol	13.4120	3.0106	194.7	197.9	−3.2
22	4-nonanol	13.4120	3.0474	193.0	196.0	−3.0
23	5-nonanol	13.4120	3.0580	195.1	195.4	−0.3
24	2-me-1-propanol	4.5000	2.6351	107.9	103.7	4.2
25	2-me-2-propanol	4.5000	2.9356	82.4	87.5	−5.1
26	2-me-1-butanol	6.4444	2.6884	128.7	125.8	2.9
27	2-me-2-butanol	6.4444	3.0353	102.0	107.1	−5.1
28	3-me-1-butanol	6.4444	2.6420	131.2	128.3	2.9
29	3-me-2-butanol	6.4444	2.8850	111.5	115.2	−3.7
30	2-me-1-pentanol	7.9167	2.7216	148.0	142.9	5.1
31	3-me-1-pentanol	8.2639	2.6752	152.4	149.9	2.5
32	4-me-1-pentanol	7.9167	2.6551	151.8	146.5	5.3
33	2-me-2-pentanol	7.9167	3.0885	121.4	123.2	−1.8
34	3-me-2-pentanol	8.2639	2.9383	134.2	135.7	−1.5
35	4-me-2-pentanol	7.9167	2.8919	131.7	133.8	−2.1
36	2-me-3-pentanol	7.9167	2.9846	126.6	128.8	−2.2
37	3-me-3-pentanol	8.2639	3.1349	122.4	125.1	−2.7
38	2-me-2-hexanol	9.6739	3.1217	142.5	144.0	−1.5
39	3-me-3-hexanol	9.8161	3.1882	142.4	142.2	0.2
40	7-me-1-octanol	12.9433	2.6861	206.0	209.4	−3.4
41	2-et-1-butanol	8.2639	2.7417	146.5	146.3	0.2
42	3-et-3-pentanol	9.9583	3.2345	142.5	141.5	1.0
43	2-et-1-hexanol	11.5178	2.7975	184.6	185.1	−0.5
44	2,2-dime-1-propanol	5.0000	2.7347	113.1	104.8	8.3
45	2,2-dime-1-butanol	7.1667	2.7880	136.8	129.7	7.1
46	2,3-dime-1-butanol	7.8889	2.7417	149.0	141.5	7.5
47	3,3-dime-1-butanol	7.1667	2.6953	143.0	134.7	8.3
48	2,3-dime-2-butanol	7.8889	3.1349	118.6	120.3	−1.7
49	3,3-dime-2-butanol	7.1667	2.9846	120.0	119.1	0.9
50	2,3-dime-2-pentanol	9.5833	3.1882	139.7	139.2	0.5
51	3,3-dime-2-pentanol	9.2083	3.0379	133.0	142.5	−9.5
52	2,2-dime-3-pentanol	8.5139	3.0843	136.0	131.1	4.9
53	2,4-dime-3-pentanol	8.8889	3.0843	138.8	135.9	2.9
54	2,6-dime-4-heptanol	12.3061	3.0982	178.0	179.0	−1.0
55	2,3-dime-3-pentanol	9.5833	3.2345	139.0	136.7	2.3
56	3,5-dime-4-heptanol	12.7922	3.1908	187.0	180.3	6.7
57	2,2,3-trime-3-pentanol	10.4028	3.3342	152.2	141.9	10.3
58	3,5,5-trime-1-hexanol	11.4206	2.7433	193.0	186.8	6.2

**Table 6.** Experimental and calculated water solubility (lg *W*), *n*-octanol/water partition coefficients (lg *P*_OW_) of 58 saturated alcohols and the topological descriptors values used in the QSPR models.

**No.**	**Alcohol**	**MPEI**	**SX_1CH_**	**lg*W*(Exp.)**	**lg*W*(Cal.)**	**lg*P*_OW_(Exp.)**	**lg*P*_OW_(Cal.)**
1	1-butanol	2.5887	−6.5340	−0.03	0.00	0.84	0.75
2	2-butanol	2.7854	−6.5407	−0.39	−0.25	0.61	0.61
3	2-methyl-1-propanol	2.6348	−6.5407	−0.10	−0.05	0.61	0.72
4	1-pentanol	2.6219	−7.5404	0.59	0.56	1.34	1.28
5	3-methyl-1-butanol	2.6420	−7.5453	0.51	0.54	1.14	1.27
6	2-methyl-1-butanol	2.6884	−7.5440	0.46	0.48	1.14	1.23
7	2-pentanol	2.8386	−7.5453	0.28	0.28	1.14	1.13
8	3-pentanol	2.8850	−7.5440	0.21	0.22	1.14	1.09
9	3-methyl-2-butanol	2.8850	−7.5496	0.21	0.22	1.14	1.10
10	2-methyl-2-butanol	3.0353	−7.5706	0.23	0.04	0.89	1.00
11	2,2-dimethyl-1-propanol	2.7347	−7.5706	0.30	0.43	1.36	1.22
12	1-hexanol	2.6446	−8.5424	1.21	1.13	1.84	1.82
13	2-hexanol	2.8718	−8.5469	0.87	0.84	1.61	1.66
14	3-hexanol	2.9383	−8.5434	0.80	0.75	1.61	1.61
15	3-methyl-3-pentanol	3.1028	−8.5480	0.39	0.54	1.39	1.49
16	2-methyl-2-pentanol	3.0886	−8.5515	0.51	0.56	1.39	1.51
17	2-methyl-3-pentanol	2.9846	−8.5454	0.70	0.69	1.67	1.58
18	3-methyl-2-pentanol	2.9383	−8.5454	0.71	0.75	1.67	1.61
19	2,2-dimethyl-1-butanol	2.7880	−8.5480	1.04	0.94	1.57	1.72
20	2,3-dimethyl-1-butanol	2.7417	−8.5454	0.50	1.00	1.57	1.75
21	2,3-dimethyl-2-butanol	3.1349	−8.5526	0.37	0.50	1.17	1.47
22	3,3-dimethyl-2-butanol	2.9846	−8.5526	0.64	0.69	1.19	1.58
23	2-methyl-1-pentanol	2.7216	−8.5434	1.05	1.03	1.78	1.76
24	4-methyl-1-pentanol	2.6551	−8.5469	0.99	1.12	1.78	1.81
25	4-methyl-2-pentanol	2.8919	−8.5486	0.81	0.81	1.67	1.64
26	2-ethyl-1-butanol	2.7417	−8.5400	1.21	1.00	1.78	1.75
27	1-heptanol	2.6611	−9.5424	1.81	1.70	2.34	2.36
28	2-heptanol	2.8945	−9.5454	1.55	1.40	2.31	2.19
29	3-heptanol	2.9715	−9.5414	1.39	1.30	2.31	2.14
30	4-heptanol	2.9916	−9.5392	1.39	1.27	2.31	2.12
31	2-methyl-2-hexanol	3.1217	−9.5480	1.07	1.11	1.84	2.03
32	5-methyl-2-hexanol	2.9050	−9.5482	1.38	1.39	2.19	2.19
33	3-methyl-2-hexanol	3.1882	−9.5405	0.98	1.02	1.87	1.98
34	2-methyl-3-hexanol	3.0058	−9.5407	1.32	1.25	2.19	2.11
35	2,2-dimethyl-1-pentanol	2.8212	−9.5405	1.52	1.49	2.39	2.24
36	2,4-dimethyl-1-pentanol	2.7548	−9.5432	1.60	1.58	2.19	2.29
37	4,4-dimethyl-1-pentanol	2.6883	−9.5480	1.55	1.67	2.39	2.34
38	2,3-dimethyl-2-pentanol	3.1882	−9.5556	0.91	1.03	2.27	1.99
39	2,4-dimethyl-2-pentanol	3.1419	−9.5487	0.93	1.08	1.67	2.02
40	2,2-dimethyl-3-pentanol	3.0843	−9.5556	1.16	1.16	2.27	2.06
41	2,3-dimethyl-3-pentanol	3.2345	−9.5399	0.84	0.96	1.67	1.95
42	2,4-dimethyl-3-pentanol	3.0843	−9.5409	1.32	1.15	2.31	2.06
43	1-octanol	2.6736	−10.5390	2.35	2.28	2.84	2.90
44	2-octanol	2.9110	−10.5423	2.07	1.97	2.84	2.73
45	2-ethyl-1-hexanol	2.7975	−10.5296	2.17	2.11	2.84	2.81
46	1-nonanol	2.6820	−11.5348	3.00	2.86	3.57	3.45
47	2-nonanol	2.9235	−11.5372	2.74	2.55	3.36	3.28
48	3-nonanol	3.0106	−11.5315	2.66	2.43	3.36	3.21
49	4-nonanol	3.0474	−11.5280	2.59	2.38	3.36	3.18
50	5-nonanol	3.0580	−11.5268	2.49	2.37	3.36	3.17
51	2,6-dimethyl-4-heptanol	3.0982	−11.5273	2.51	2.32	3.31	3.15
52	1-decanol	2.6892	−12.5296	3.70	3.44	4.01	3.99
53	2-undcanol	2.9391	−13.5220	2.94	3.71	4.42	4.36
54	1-dodecanol	2.7011	−14.5138	4.80	4.61	5.06	5.08
55	1-tetradecanol	2.7098	−16.4948	5.52	5.77	6.11	6.17
56	1-pentadecanol	2.7132	−17.4838	5.84	6.36	6.64	6.71
57	1-hexadecanol	2.7163	−18.4720	7.00	6.94	7.17	7.26
58	1-octadecanol	2.7214	−20.4476	8.40	8.11	8.22	8.35
